# Long-range regulatory interactions at the 4q25 atrial fibrillation risk locus involve *PITX2c* and *ENPEP*

**DOI:** 10.1186/s12915-015-0138-0

**Published:** 2015-04-17

**Authors:** Luis A Aguirre, M Eva Alonso, Claudio Badía-Careaga, Isabel Rollán, Cristina Arias, Ana Fernández-Miñán, Elena López-Jiménez, Amelia Aránega, José Luis Gómez-Skarmeta, Diego Franco, Miguel Manzanares

**Affiliations:** Centro Nacional de Investigaciones Cardiovasculares (CNIC), Melchor Fernández Almagro 3, 28029 Madrid, Spain; Centro Andaluz de Biología del Desarrollo (CABD), CSIC-Universidad Pablo de Olavide-Junta de Andalucía, ctra. de Utrera km1, 41013 Seville, Spain; Department of Experimental Biology, Faculty of Experimental Sciences, University of Jaen, Paraje de las Lagunillas s/n, 23071 Jaén, Spain

**Keywords:** Atrial fibrillation, Chromosome conformation, *ENPEP*, *PITX2*, Regulatory element

## Abstract

**Background:**

Recent genome-wide association studies have uncovered genomic loci that underlie an increased risk for atrial fibrillation, the major cardiac arrhythmia in humans. The most significant locus is located in a gene desert at 4q25, approximately 170 kilobases upstream of *PITX2,* which codes for a transcription factor involved in embryonic left-right asymmetry and cardiac development. However, how this genomic region functionally and structurally relates to *PITX2* and atrial fibrillation is unknown.

**Results:**

To characterise its function, we tested genomic fragments from 4q25 for transcriptional activity in a mouse atrial cardiomyocyte cell line and in transgenic mouse embryos, identifying a non-tissue-specific potentiator regulatory element. Chromosome conformation capture revealed that this region physically interacts with the promoter of the cardiac specific isoform of *Pitx2*. Surprisingly, this regulatory region also interacts with the promoter of the next neighbouring gene, *Enpep*, which we show to be expressed in regions of the developing mouse heart essential for cardiac electrical activity.

**Conclusions:**

Our data suggest that de-regulation of both *PITX2* and *ENPEP* could contribute to an increased risk of atrial fibrillation in carriers of disease-associated variants, and show the challenges that we face in the functional analysis of genome-wide disease associations.

**Electronic supplementary material:**

The online version of this article (doi:10.1186/s12915-015-0138-0) contains supplementary material, which is available to authorized users.

## Background

Genome-wide association studies (GWAS) have exponentially increased our knowledge of the genetic component of human disorders, revealing unsuspected loci that harbour variants linked to an increased risk of disease [1]. However, the majority of GWAS signals fall in non-coding regions of the genome, which has made their functional analysis particularly challenging [2,3]. Even the identification of the genes targeted by disease-associated variants is not straightforward, as mere proximity can result in incorrect identification of the culprit gene [4].

Atrial fibrillation (AF) is the most common cardiac arrhythmia in humans [5] although its pathophysiologic basis is still not clearly understood, presenting a challenge for cardiovascular research and therapy. AF is defined as a supraventricular tachyarrhythmia characterised by uncoordinated atrial activation, and is frequently observed as a consequence of various systemic and cardiac disorders (syndromic AF) [6]. However, in 10% to 20% of cases AF is not associated with other cardiovascular disease, and thus is dubbed ‘idiopathic’ or ‘lone’ AF that mostly occurs in patients under 60. The strong association of AF onset with risk factors, such as age, sex, ethnicity, hypertension and other heart diseases [7], originally suggested it being a non-genetic disorder [8]. Nevertheless, in the last two decades several epidemiological studies pointed to a significant incidence of genetic factors [9]. Furthermore, rare mutations in a dozen genes, mostly encoding ion channel subunits [8], are associated with AF as part of wider cardiac electrical syndromes.

At least ten loci have been linked to AF by GWAS in large cohorts of non-related patients of distinct ethnic backgrounds [10-12]. The most highly AF associated variants identified in all studies are located on chromosome 4q25 [11], 170 kilobases (kb) distal to *PITX2* and within a 1.5 megabases (Mb) intergenic gene desert. *PITX2* encodes an evolutionarily conserved homeodomain transcription factor that is involved in the establishment of left-right asymmetry and cardiovascular development in the vertebrate embryo. In mice and humans the *PITX2* gene generates several isoforms. PITX2a and PITX2b are alternative splicing variants produced from a common promoter, whereas PITX2c is the product of an alternative promoter and is the main isoform expressed in the heart [13].

Mouse *Pitx2c* is first expressed in the left lateral plate mesoderm of the early embryo, as part of the network regulating the establishment of left-right asymmetry during development [14], and is then expressed in the left side of the heart at early stages [15]. At later stages, expression follows a dynamic pattern, being present in the left atrium, the myocardium sleeves of the pulmonary veins, the atrio-ventricular cushions or the base of the ventricles [16,17]. Germline homozygous deletion of *Pitx2* results in embryonic lethality and numerous cardiac malformations, such as right atrial isomerism and outflow tract defects, varying from double outlet right ventricle or transposition of the great arteries to persistent truncus arteriosus [18]. More recent research showed that *Pitx2*c is expressed in adult mice and human hearts, predominantly in the left atrium (levels in the right atrium and the ventricles are 100-fold lower), and that its levels of expression decrease in atria of AF patients [13,19]. Furthermore, *Pitx2*c heterozygous or atrial specific deletion of *Pitx2c* display molecular and physiological hallmarks of human AF [13,19,20], which also is observed when *Pitx2* is deleted in adult mice [21]. Altogether, these data support the hypothesis that *PITX2* could play a causal role in the pathogenesis of AF and that its function could be altered by genomic elements located in the vicinity of the single nucleotide polymorphisms (SNP) in 4q25 that correlate with of AF.

In this study, we explored whether the 4q25 region spanning the AF-associated variants identified by GWAS harbours putative regulatory elements that could be acting on neighbouring genes. By tissue culture, *in vivo* transgenics and analysis of chromatin structure, we have found that this region contains potentiator cis-regulatory elements that interact with the promoters of *Pitx2c* and, unexpectedly, *Enpep*, the next gene located downstream of *Pitx2*. Given the expression of *Enpep* in the sinoatrial node (SAN) and the co-expression of *Pitx2* and *Enpep* in pro-arrhythmogenic regions of the embryonic heart, such as the sleeves of the pulmonary veins, our data suggest that de-regulation of these genes could underlie increased risk of AF.

## Results

### Genomic analysis of the 4q25 AF-associated locus

**Figure 1:**
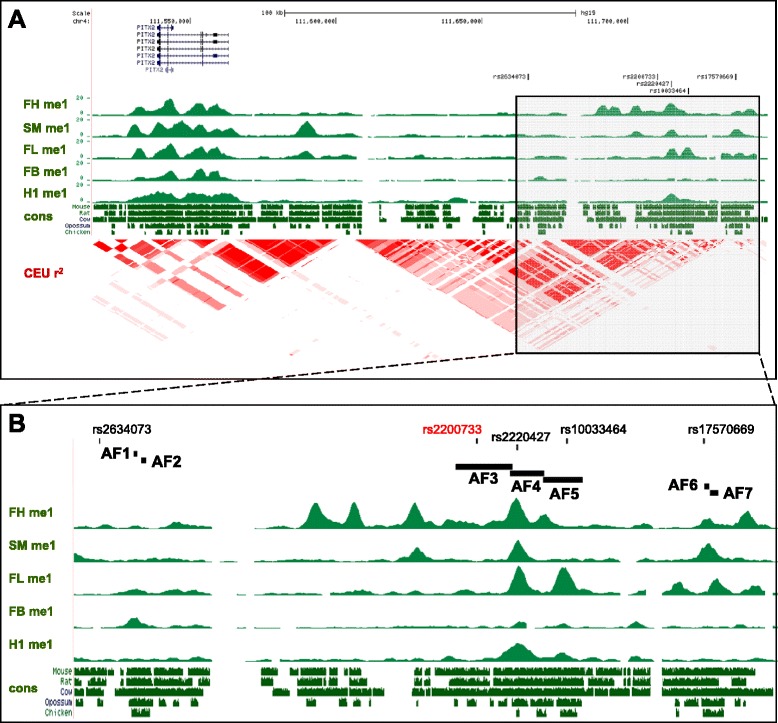
Genomic landscape of the atrial-fibrillation associated region 4q25. **(A)** A 230 kb view of the 4q25 (hg19; chr4:111,516,448-111,747,857) gene desert, showing the position of AF risk-associated SNPs (vertical black bars) distal to *PITX2*. The different *PITX2* isoforms of the gene are shown above with *PITX2c*, the main cardiac isoform, on top. Below, UCSC tracks of the region showing the distribution of H3K4me1 marks (me1) in foetal heart (FH), smooth muscle (SM), foetal liver (FL), foetal brain (FB) and human embryonic stem cells (H1); the conservation (cons) between human and mouse, rat, cow, opossum and chicken; and the linkage disequilibrium structure from the HapMap Project (CEU r^2^). **(B)** A 85 kb zoom of the shaded rectangle shown in **A** (hg19; chr4:111,662,786-111,747,668) indicating the fragments (AF1-AF7) tested for regulatory activity in this study. The lead AF risk associated SNP rs2200733 is highlighted in red. AF, atrial fibrillation; UCSC, University of California Santa Cruz genome browser.

To identify putative cis-regulatory elements located in the 4q25 region we analysed the evolutionary conservation [22] and distribution of histone modifications associated with active elements [23] (H3K4me1) in an 85 kb window containing the main SNPs that have been associated with an increased risk of AF by GWAS (Figure 1). This window is centred on the lead rs2200733 SNP [11] that has repeatedly been identified as the most significant variant associated with AF [10-12,24-26], and spans a region that includes other distal SNPs (rs2634073 and rs17570669) that lie in the proximity of sequences highly conserved between human and mouse (Figure 1A). The region including the majority of AF-associated SNPs in 4q25 is confined to a linkage disequilibrium (LD) block [11], separated from that containing the coding exons of the gene and from adjacent LD blocks in the 1.5 Mb gene desert located centromeric to *PITX2* (Figure 1A). It is also noteworthy that the selected SNPs and the *PITX2* gene are contained together in a single topologically associated domain (TAD; Additional file 1A), as defined by Hi-C in three different human cell lines [27].

We selected seven genomic fragments (AF1-7) for further analysis (Figure 1B). Fragments AF1 and AF2 lie in close proximity to rs2634073 and show high evolutionary conservation. These fragments are included in a region (hs930) tested as part of a large scale screen for tissue specific human enhancers by transgenesis in the mouse embryo [28] and drive reporter expression in the nervous system and limbs but not in the heart. This region was also tested in transgenic zebrafish, driving expression in similar patterns but again not in the developing heart [29]. AF3 to AF5 are a set of overlapping fragments that include the lead rs2200733 variant and other highly associated SNPs, in a region with high conservation among placental mammals and H3K4me1 marks of active regulatory elements. Finally, AF6 and AF7 map to a region conserved in vertebrates including rs17570669, which has been associated with AF but is independent of rs2200733 [12].

### The 4q25 AF-associated locus contains active regulatory elements

**Figure 2:**
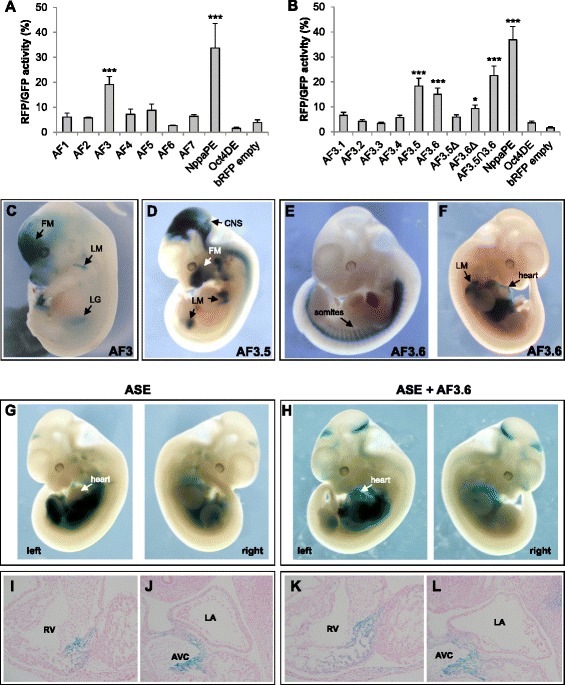
Regulatory activity of 4q25 genomic elements in cultured HL-1 atrial cardiomyocytes and in transgenic mouse embryos. **(A)** HL-1 transfection assays with the 4q25 fragments AF1 to AF7 show that only AF3 exhibits regulatory activity (*P* = 0.0004) as compared with the positive (NppaPE) and negative (Oct4DE, bRFP) controls. **(B)** Transfection assays of AF3.1 to AF3.6 overlapping fragments; only AF3.5 (*P* = 0.0002) and AF3.6 (*P* = 0.0006) show significant activity. Deletion of the overlapping fragment between them, which itself shows strong activity (AF3.5∩3.6; *P* = 0.00004), abrogates activity of AF3.5 (AF3.5Δ) but not of AF3.6 (AF3.6Δ; *P* = 0.02). **(C)** AF3 drives *lacZ* reporter expression in E13.5 transgenic mice embryos to different sites, such as the facial mesenchyme (FM), limb muscles (LM), and the left gonad (LG). **(D**-**F)** Activity in embryonic day **(E)** 10.5 to 11.5 transgenic embryos of AF3.5 **(D)** and AF3.6 **(E,** **F)** is highly variable, driving expression in diverse sites, such as the central nervous system (CNS) or facial mesenchyme **(D)**, limb muscles **(D, F)**, somites **(E)**, and heart **(F)**. **(G)** Reporter activity driven by the *PITX2* ASE element is preferentially localised to the left side with weak expression in the cardiac region. **(H)** The chimeric ASE + AF3.6 construct behaves in a similar way to ASE but with increased cardiac expression. **(I**-**J)** Sections of the embryos shown in **G** and **H**, comparing the region of the right ventricle (RV) of ASE **(I)** and ASE + AF3.6 **(K)**, which shows a broader domain of reporter expression. Similarly, expression in the atrioventricular canal (AVC) is broader in ASE + AF3.6 **(L)** compared to ASE **(J)**. For **A** and **B**, data are expressed as mean ± SEM. Statistical significance versus empty pβRFP was calculated by unpaired Student’s t-test. *P <0.05, ***P <0.001. LA, left atrium. ASE, asymmetric enhancer; SEM, standard error of the mean.

We tested the regulatory activity of these fragments, corresponding to the none-risk haplotype at rs2200733 from a commercial source of human DNA, by linking them to a human minimal beta-globin promoter [30] and the reporter gene coding for monomeric red fluorescent protein (mRFP) [31]. These constructs were transfected into cultured HL-1 mouse atrial cardiomyocytes [32], a tissue culture model of the physiological conditions in which PITX2 is active [19]. As a positive control, we used a previously described enhancer from the *Nppa* gene (encoding atrial natriuretic factor; ANF) that recapitulates its endogenous expression in transgenic mice [33]; for negative controls we used the reporter constructs lacking any genomic fragment, and containing the pluripotent-specific *Oct4* distal enhancer [34]. Of the seven fragments, only AF3, which contains rs2200733 (Figure 1B), showed significant activity compared with negative controls (Figure 2A). While this could seem surprising given that other fragments show a more robust signal for histone modifications associated to regulatory elements (for example AF4, Figure 1B), enhancer prediction based on histone marks is only accurate in a fraction of cases [35].

Due to the large size of AF3 (over 7 kb), we further analysed its function by generating six overlapping fragments (AF3.1 to AF3.6) of 1 to 2 kb each (Additional file 1B). When tested in HL-1 cells, only AF3.5 and AF3.6 showed activity (Figure 2B). It is noteworthy that AF3.3, which contains rs2200733, is not active in this assay. AF3.5 and AF3.6 overlap in 80 base pairs (bp), so we then tested the activity of this minimal fragment (AF3.5∩3.6) in HL-1 cells as well as versions of AF3.5 and AF3.6 where the overlap was removed (AF3.5Δ, AF3.6Δ). AF3.5∩3.6 showed strong activity in HL-1 cells, and while AF3.5Δ was not active, AF3.6Δ retained activity although at a reduced level (Figure 2B). It is interesting to note that this 80 bp minimal fragment is highly conserved between human and mouse, and that its sequence corresponds to a short interspersed nuclear element of the MIR3 family. It has been shown that repeat sequences can act as enhancers in experimental assays [36], although their putative function *in vivo* is still under debate [37]. Therefore, we can conclude that the regulatory activity of AF3 in this assay is located in AF3.6, and that additional activity may be present in the overlapping fragment of AF3.5 and AF3.6.

Next, we assayed the activity of fragments AF3-5 and AF7 in transgenic mouse embryos, using the *lacZ* gene as a reporter. Again, only AF3 showed enhancer activity (Figure 2C, Additional file 2), confirming the results of the tissue culture assays. AF3 drives reporter expression in facial mesenchyme, limb muscles, and the left gonad, some of which are sites of expression of endogenous *Pitx2* [14,38]. Contrary to expectations, AF3 did not drive expression in the developing heart. We reasoned that regulatory elements from *PITX2* underlying the association with AF might not be active during development, and instead drive cardiac-specific expression of *PITX2* in the adult. We therefore generated transgenic mice and examined reporter expression in the heart at postnatal day 3. Again, we found no expression in cardiac tissues (Additional file 2).

When tested in mouse transgenic assays, both AF3.5 and AF3.6 showed activity (Figure 2D to F). Sites of expression include facial mesenchyme, limb muscles, somites, or pericardium, but we did not observe a reproducible pattern driven by these fragments. We can rule out the possibility that this heterogeneity is due to non-specific reporter activation as a consequence of integration site of the transgene, because of the very low percentage of *lacZ* positive embryos (out of the total number of transgenics as assessed by genotyping) obtained for genomic fragments tested showing no activity (0% to 5%) as compared to those that do (20% to 45%; Additional file 2).We also tested activity in transgenic embryos of the minimal AF3.5∩3.6 fragment, finding that it was not active (1 weak *lacZ*+ embryo out of 17 transgenics; Additional file 2).

### The 4q25 regulatory elements show non-specific potentiator activity

The above results suggest that these 4q25 elements, while they have regulatory potential, do not confer tissue specificity. To test this hypothesis further, we transfected fragments AF3, AF3.5 and AF3.6 into two cell types unrelated to the cardiac lineage: the mouse teratocarcinoma-derived pluripotent cell line P19 and human embryonic kidney (HEK) cells. We found that all three fragments were active in both cell types, closely matching the degree of activation in HL-1 cells (Additional file 3). As expected, the *Oct4*-DE was active in P19 but not in HEK cells; in contrast, the *Nppa* enhancer was not active in P19 but showed activity in HEK cells, as expected given the endogenous expression of *NPPA* in human kidney [39]. Overall, our results suggest that the regulatory elements detected in 4q25 do not act as cell type-specific enhancers, but rather as accessory elements that can potentiate the activity of tissue-specific enhancers located elsewhere in the locus.

To further prove the putative potentiator activity of 4q25 elements, we assessed the effect of AF3.6 on the activity of a previously identified intronic enhancer from *Pitx2*, which drives left-sided expression in the embryo [40]. This asymmetric enhancer (ASE) is evolutionarily conserved in sequence and function, but it is noteworthy that the ASE from human *PITX2* only drives weak expression in the mice heart compared with its mouse homologue [41]. We generated a chimeric construct containing both human AF3.6 and ASE and compared its activity to that of ASE alone in transgenic mouse embryos at 10.5 (Figure 2G-L). We first observed that when using the chimeric ASE + AF3.6 construct, the variability associated with AF3.6 alone is lost, and all embryos show the characteristic left-sided expression described for the ASE (Figure 2G, H). Importantly, we found that there is no additive effect of both genomic fragments as that observed when placing together different enhancers in the same transgenic construct [42]. In fact, we observed that adding AF3.6 to the ASE apparently increased the levels of reporter expression in the cardiac region (Figure 2G, H), as seen in sections where domains of reporter activity in the right ventricle and in the atrio-ventricular canal are expanded in ASE + AF3.6 embryos compared to ASE (Figure 2I-L). When we examined in detail reporter expression in the developing heart for all transgenic embryos, we found that AF3.6 increases the number of embryos expressing lacZ in the left atrium (two out of five for ASE, as compared to seven out of ten for ASE + AF3.6; Additional file 4). The results of these assays further suggest that 4q25 elements have an accessory role in defining *PITX2* expression acting in conjunction with other regulatory elements.

### The three-dimensional architecture of the *Pitx2* locus identifies promoter-specific long range interactions

**Figure 3:**
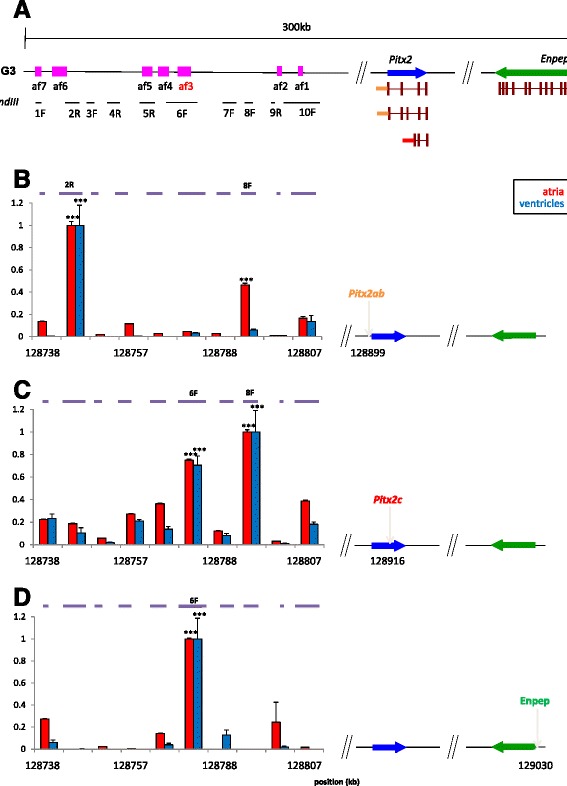
Long-range chromatin interactions in the mouse *Pitx2* genomic locus. **(A)** Schematic representation of a 300 kb region of the mouse genome syntenic to human 4q25. The approximate locations of regions orthologous to human AF1-AF7 are indicated by pink boxes (af1 to af7; af3, in red, is orthologous to human AF3, which contains the lead SNP rs2200733). The promoter regions from which anchor primers for 3C were generated are indicated (*Pitx2ab*, blue; *Pitx2c*, red; *Enpep*, green). Genomic *Hin*dIII fragments tested for their interaction with anchor promoter primers are represented by black horizontal bars (1 to 10; F and R denote primer design). **(B-D)** Normalised 3C interactions, expressed as crosslinking frequencies (y-axis), between the test fragments and the promoters of *Pitx2ab* **(B)**, *Pitx2c* **(C)** and *Enpep* **(D)** in atria (red) and ventricle (blue). In each graph, the highest crosslinking frequency values were set to 1. Genome coordinates (x-axis) are from the mouse NCBI37/mm9 assembly. Statistical significance was assessed following one-way ANOVA test of Student-Newman-Keuls. ***P <0.001. Error bars represent ± SEM. ANOVA, analysis of variance; SEM, standard error of the mean; 3C, chromosome conformation capture.

Although the above evidence shows that 4q25 includes regulatory elements, there is no direct evidence that this genomic region acts on *PITX2* or that, if it does, it shows any specificity regarding the cardiac and non-cardiac isoforms produced from two alternative promoters. To answer these questions, we analysed the three-dimensional organisation of the locus by chromosome conformation capture (3C) [43,44], to address if these regions physically contact the promoters of *PITX2*. Because we aimed to perform the assays in the physiological context of the heart, we selected the mouse genome region syntenic to human 4q25 and analysed chromatin from the atria and ventricles of adult mouse hearts. Based on the relative order and position of conserved sequence blocks between human and mouse, we could unambiguously map mouse fragments (af1-af7) equivalent to the human AF1-AF7 fragment series tested above (Figure 3A).

We probed the interaction of *Hin*dIII restriction fragments containing the *Pitx2a,b* or *Pitx2c* promoter with ten fragments spanning over 100 kb of the distal region on mouse chromosome 3 syntenic to the AF-associated 4q25 locus in humans (Figure 3A). Using atria and ventricles from adult mice we observed a clear pattern of long range interactions, with regions interacting specifically with the *Pitx2a,b* promoter (fragment 2R; Figure 3B), the *Pitx2c* promoter (fragment 6F; Figure 3C), or with both (fragment 8F; Figure 3B, C). The latter result prompted us to ask if fragment 8F had regulatory activity in HL-1 cells, since it is not included in any of the previously tested fragments. This was not the case, suggesting that this genomic region has other architectural roles in configuring the regulatory landscape of *Pitx2*. To further examine the specificity of chromatin interaction between the AF-associated region and *Pitx2c*, we checked the interaction of a fragment containing the promoter of *Enpep*, the next neighbouring gene distal to *Pitx2* in both mouse and humans (Figure 3A). To our surprise, we found a robust interaction between fragment 6F and *Enpep* (Figure 3D), suggesting that the 4q25 regulatory landscape is partially shared between *PITX2* and *ENPEP*. The specificity of the interactions of fragments 2R, 6F and 8F with *Pitx2ab*, *Pitx2c* and *Enpep* was tested by using a series of control primers located upstream and downstream of the promoters, which showed no interactions (Additional file 5).

**Figure 4:**
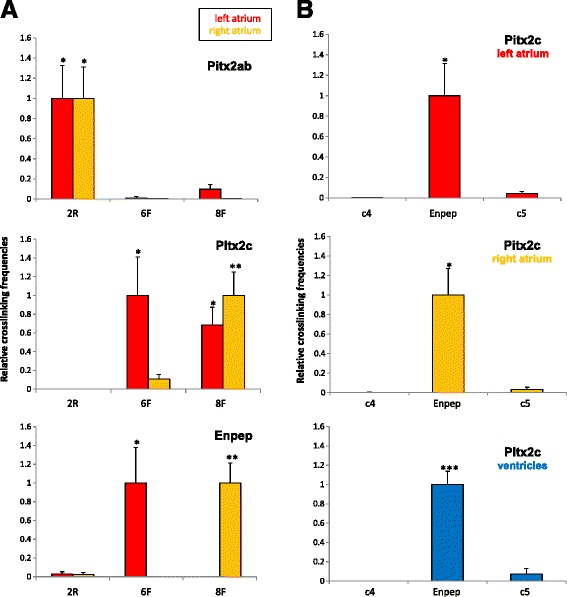
Differential chromatin interactions of *Pitx2* and *Enpep* in left and right atrium. **(A)** Normalised 3C interactions, expressed as crosslinking frequencies (y-axis), between fragments 2R, 6F and 8F, and the promoters of *Pitx2ab*, *Pitx2c* and *Enpep*, in left (red) and right (yellow) atrium. **(B)** 3C interaction between the *Pitx2c* and *Enpep* promoters, including control regions upstream (c4) and downstream (c5) of *Enpep* (see Additional file 5), in left (red) and right (yellow) atrium, as well as in ventricles (blue). In each graph, the highest crosslinking frequency values were set to 1. Statistical significance was assessed following one-way ANOVA test of Student-Newman-Keuls. *P <0.05, **P <0.01, ***P <0.001. Error bars represent ± SEM. ANOVA, analysis of variance; SEM, standard error of the mean; 3C, chromosome conformation capture.

We next asked if the interactions we observed showed regional differences in atria, as AF constitutes a disorder of the left atrium and this is the region where *PITX2* is prominently expressed [13]. While fragment 2R interacted specifically with the *Pitx2ab* promoter in both left and right atrium, fragment 6F interacts only in left atrium with *Pitx2c* and *Enpep*. On the other hand, fragment 8F shows interaction with *Pitx2c* in both atria but only in the right atrium with *Enpep* (Figure 4A). Given the fact that the promoters of *Pitx2c* and *Enpep* share many of the interactions tested, we examined if they were physically associated and if this was region-specific. We found a robust and specific promoter-promoter interaction in both atria and in ventricles (Figure 4B), therefore independent of transcription and in line with recent observations on the role of pre-existing promoter-promoter interactions for structuring the genome [45].

It should be noted that fragment 6F contains the region conserved with human fragment AF3, thereby suggesting that the region with regulatory activity and that contains the lead SNP associated with AF (rs2200733) interacts in a specific manner with the promoters of the cardiac-specific isoform of *Pitx2* and the neighbouring gene, *Enpep*. The 3C analysis of the mouse *Pitx2/Enpep* locus thus revealed an unexpected complexity of specific and shared chromatin interactions between the regions containing the potentiator elements and the different promoters studied that could be related to their function.

### *Enpep* is expressed in arrhythmogenic sites in the embryonic heart

**Figure 5:**
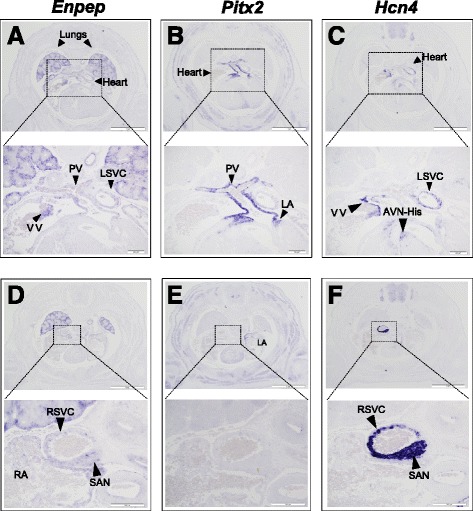
*Enpep* is expressed in the embryonic mouse heart. **(A to F)** Expression of *Enpep* **(A, D)**, *Pitx2* **(B, E)** and *Hcn4* **(C, F)** in E14.5 mouse embryos shown by *in situ* hybridization on two sets of consecutive sections (**A to C** **and** **D to F**). In addition to strong expression in the endothelial lining of the lungs, *Enpep* is expressed in a restricted pattern in the heart **(A, D)**, where it is co-expressed with *Pitx2* in the pulmonary veins (PV; zoom in **A** and **B**) and with *Hcn4* in the leaflet of the venous valve (VV) and left superior vena cava (LSVC; zoom in **A** and **C**) as well as in the right superior vena cava (RSVC) and sinoatrial node (SAN; zoom in **D** and **F**). It is noteworthy that *Enpep* is not expressed in the myocardium of the left atria (LA), as is *Pitx2* (zoom in **B** and **E**). AVN-His, atrial ventricular node-bundle of His; RA, right atria. Scale bars, 1 mm; close-ups, 200 μm. E, embryonic day.

*ENPEP* encodes aminopeptidase A, which cleaves angiotensin II to produce angiotensin III as part of the renin-angiotensin system [46]. Therefore ENPEP is involved in the control of blood pressure, and accordingly it is expressed in the renal system and endothelial cells, and knockout mice for *Enpep* develop hypertension [47]. However, at present there is no report for expression or a role of *Enpep* in the heart. In light of our results, we examined the expression of *Enpep* in the E14.5 mouse embryos by *in situ* hybridization on tissue sections (Figure 5). *Enpep* is strongly expressed in the endothelial lining of the lungs, but also in a specific and restricted pattern in the developing heart (Figure 5A, D). We compared *Enpep* expression with that of *Pitx2* (Figure 5B, E) and *Hcn4* (Figure 5C, F), which encodes a voltage-gated ion channel and at this stage is a marker of most of the cardiac conduction system [48,49]. This analysis showed that *Enpep* is co-expressed with *Pitx2* in the pulmonary veins but not in the myocardium of the left atrium (Figure 5A, B, D, E), and is co-expressed with *Hcn4* in the left and right superior venae cavae and in the SAN (Figure 5A, C, D, F). *Enpep* is thus expressed in the embryonic mouse heart in key components of the cardiac conduction system such as the SAN. Moreover, *Enpep* is also expressed at the base of the pulmonary veins and the junction of the caval veins, regions prone to initiate ectopic electrical beats, which lead in many cases to the onset of AF [7].

## Discussion

The advent of GWAS has radically changed our perspective on the genetic analysis of common diseases in humans. On the one hand, a plethora of novel loci linked to increased disease risk have been uncovered, which await further analysis before possible translation to the clinic [2]. On the other hand, the vast majority of risk variants are located in non-coding genomic sequences, pointing to a fundamental role for variation in cis-regulatory elements as the basis of common diseases [50,51]. Understanding the role and function of these genomic elements will be fundamental to making the most of the discoveries of GWAS.

The genomic analysis of AF is a prime example in this regard. All GWAS carried out to date have shown that the major loci for AF lie in an intergenic gene desert in 4q25, located distal to the developmental regulator *PITX2* [10-12]. Despite its early role in establishing the left-right patterning of the heart and its prominent expression in the left atrium, no evidence suggested a role for *PITX2* in the pathophysiology of AF [9]. Subsequent analysis of loss-of-function mouse models of *Pitx2* have confirmed that it plays a pivotal role in regulating different atrial phenotypes by distinguishing electrical from working myocardium in the right and left atria respectively [13,19,20]. However, no reports to date have provided evidence as to how distal variants in 4q25 act on *PITX2*. Even more surprisingly, a recent report showed that 4q25 variants do not correlate with *PITX2* expression in atrial tissue from human patients [52]. This evidences that variants identified by GWAS may have extremely subtle effects, which fall below the threshold of detection of current analytical tools and approaches.

Our analysis of the regulatory structure of the 4q25 locus shows that genomic sequences in close proximity to AF-linked variants can act as transcriptional regulatory elements both in tissue culture and in mouse embryos. Rather unexpectedly, and contrasting with other cases of GWAS-related enhancers in the cardiovascular system [53,54], these elements are not specific to cardiac cell types, either in culture or *in vivo*. The 4q25 elements show equal activity when transfected into cell types of different origin. Furthermore, in transgenic mouse embryos these elements drive highly variable patterns of reporter expression. These results suggest that the cis-regulatory elements in 4q25 do not act as classical tissue-specific enhancers, but as potentiator elements that would act in cooperation with elements located elsewhere in the locus that dictate tissue restricted expression. In the case of mouse *Pitx2*, an intronic enhancer (ASE) has been described that drives left-side specific expression in the early embryo and later in the heart, liver and other organs [41]. It is conceivable that precise control of spatial and quantitative expression of *PITX2* requires interplay of the ASE and the 4q25 potentiator. In fact, when placed together and tested by transgenics, the variability of 4q25 elements is lost and it can modulate the activity of ASE. It is possible that this potentiator could also modulate the activity of other yet to be identified regulatory elements from the locus.

By analysing the physical interaction between promoters and intergenic sequences of the mouse region syntenic to 4q25, we have found a further degree of complexity in the chromatin structure of the region. First, there is a clear specificity in the interaction of distal elements with the alternative promoters of the different *Pitx2* isoforms, despite their being separated by less than 10 kb. In this regard, it is noteworthy that the region containing the potentiator activity we have described interacts specifically with the promoter of the cardiac-specific *Pitx2c* isoform. Furthermore, this interaction occurs specifically in the left atrium. These results provide additional support to the specific role of the region identified by GWAS in regulating *PITX2* in the heart.

More surprising was the fact that this same region physically interacts with the promoter of *Enpep*, the neighbouring gene located distal to *Pitx2*. This opens the possibility that *Enpep* could also be a transcriptional target of the identified cis-regulatory elements. ENPEP, as part of the renin-angiotensin system, has been shown to control blood pressure, and hypertension is a known risk factor for AF [55]. However, 4q25 variants are associated with lone AF, with no co-occurrence of hypertension [25], and independent variants located in the proximity of *ENPEP* but not in the 4q25 AF loci are associated with changes in blood pressure [56]. Furthermore, there is no reported correlation between expression of *ENPEP* and 4q25 variants in the blood or adipose tissue [11]. We can therefore conclude that the possible regulation of *ENPEP* by the 4q25 potentiator elements would be unrelated to its known role in the control of blood pressure. Our re-evaluation of *Enpep* expression in the developing mouse heart by *in situ* hybridization reveals co-expression with *Pitx2* in the pulmonary veins, a region with pro-arrhythmogenic potential [57], and in the SAN of the right atria, a key component of the cardiac conduction system where the electrical impulse is generated. Our preliminary observations suggest that incorrect regulation of *ENPEP* in these locations could be linked to AF. The precise role of ENPEP in the heart remains to be identified and could offer novel insight into the pathogenesis of AF.

## Conclusions

We have shown that novel cis-regulatory elements are located in the region of 4q25 associated with an increased risk for AF. These elements establish complex long-distance interactions with the promoters of both *Pitx2c* and *Enpep*, and therefore could regulate the transcription of these genes. A potential limitation of our study is the fact that while we have used human genomic DNA for regulatory assays, the chromatin structure of the *Pitx2*/*Enpep* locus and the expression of *Enpep* in the heart was carried out in mouse. However, the sequence conservation in the regions studied, as well as conserved synteny of the locus and of gene functions strongly suggests that regulatory mechanisms will also be conserved between human and mouse. Overall, our results suggest that de-regulation of either one or both *PITX2* and *ENPEP* might have a causal role in the development of AF. Future work will be needed to identify the causal variants and the upstream regulatory factors that act through the potentiator elements described here.

Our study also highlights the challenges we face in the functional analysis of genetic variation identified by GWAS. Our understanding of the nature and function of non-coding genomic elements is still incomplete, despite the wealth of genome-wide data available through ENCODE and similar projects [58,59]. We are greatly limited by the breadth and specificity of available assays to interrogate the function of a DNA fragment. We can hypothesise that only a fraction of GWAS hits will represent classical tissue-specific enhancers, whose characterisation is feasible with current tools. Many cases will affect other regulatory elements with not such a clear-cut and easily identifiable role in gene transcription, such as potentiators or modulators (as we have identified here), but also silencers, insulators or stabilisers. Novel tools and assays will need to be devised to fully understand the regulatory variation underlying common human disease.

## Methods

### Cloning

Commercial Clontech (Mountain View, California, USA) human DNA was used for PCR amplification of all the tested genome fragments from chromosome 4q25 (for primers used see Additional file 6). We used the pGem-T Easy Promega (Madison, Wisconsin, USA) vector for initial cloning of the PCR products, followed by digestion with *NotI* New England BioLabs (Ipswich, Massachusetts, USA) and subsequent cloning in enhancer-detection vectors containing the human minimal beta-globin promoter and either monomeric red fluorescence protein (pβRFP) or *lac*Z (p1230) reporter genes.

### Cell culture and transfections

Mouse HL-1 atrial cardiomyocytes were cultured in Claycomb medium Sigma (St. Louis, Missouri, USA) supplemented with 10% (v/v) inactive (56°C, 30 minutes) fetal bovine serum (FBS) (Sigma), 4 mmol/L L-glutamine (Sigma), 100 μmol/L norepinephrine (Sigma) and 100 U/mL penicillin-streptomycin (Sigma). All seeding supports were previously coated for 24 hours with a solution of gelatin (0.02% w/v, Sigma) and fibronectin (25 μg/mL, Sigma). Mouse P19 embryonic teratocarcinoma cells (a kind gift from Christine Mummery, Leiden University Medical Center, The Netherlands) were cultivated in α-minimal essential medium (α-MEM, Gibco (Grand Island, New York, USA)) containing 10% FBS, 100 U/mL penicillin-streptomycin and 4 mmol/L L-glutamine. HEK293T human embryonic kidney cells were cultured in Dulbecco’s modified Eagle’s medium (DMEM, Sigma) supplemented with 10% FBS, 4 mmol/L L-glutamine and 100 U/mL penicillin-streptomycin.

One day before transfections, cells were counted and plated at a density of 5 × 10^5^ cells per p12 well (HL-1 cells) or p6 (P19 and HEK293T) with complete growth medium and no antibiotics. Cells were co-transfected with 2 μg of pβRFP vector containing the appropriate 4q25 fragment and 1 μg of pCAGGS-GFP (a kind gift from Joaquín Rodríguez-León, University of Extremadura, Badajoz, Spain) as an internal transfection efficiency control; co-transfections were performed with 6 μL of Lipofectamine 2000 Invitrogen (Waltham, Massachusetts, USA). Cells were transferred to complete medium with antibiotics after five hours. The empty vector pβRFP was used as a negative control.

Forty-eight hours after transfection, cultures were photographed (Zeiss) and fluorescent cells automatically counted (ImageJ) in twelve independent random fields per well (for the transfections of AF1 to AF7 in HL-1 cells; Figure 2A), or were measured by fluorescence activated cell sorting (FACS) (LSRFortessa (BD Biosciences; Franklin Lakes, New Jersey, USA) Flow Cytometer) in all other transfections. Three independent experiments with three technical replicates each were quantified in all cases. Relative regulatory enhancer activity was then calculated as the ratio of red cells (RFP^+^) to total green (GFP^+^) control cells, expressed as mean ± standard error of the mean (SEM) and statistically analysed by unpaired Student’s t-test (Prism5), with the significance threshold set at *P* <0.05.

### Transient transgenic mice

p1230-derived constructs were digested with *Sac*II and *Sal*I (New England BioLabs) to remove the plasmid backbone, and the fragment was purified using the Qiagen gel extraction kit. DNA fragments were diluted in microinjection buffer (10 mmol/L Tris–HCl, pH7.4, 0.1 mmol/L ethylenediaminetetraacetic acid (EDTA)) at 5 to 7 ng/μL and injected into zygote pronuclei obtained from crosses of (C57BL/6xCBA/J)F1 mice. Injected zygotes were transferred to CD1 foster mothers, following standard procedures [60]. At the desired stage, mice were euthanised and embryos dissected and stained for β-galactosidase activity [60]. All embryos were genotyped for *lacZ* by PCR, using primers for *Myogenin* (Additional file 6) as an internal control for calculating transgenic efficiency and the percentage of embryos expressing lacZ (Additional file 2).

Animal studies were approved by the local ethics committee. All animal procedures conformed to EU Directive 2010/63EU and Recommendation 2007/526/EC regarding the protection of animals used for experimental and other scientific purposes, enforced in Spanish law under Real Decreto 1201/2005.

### Chromosome conformation capture (3C) assays

The 3C protocol was performed essentially as described [61]. Hearts from adult (C57BL/6xCBA/J)F1 female mice were dissected into atria and ventricles. After mincing with a scalpel, tissue was mechanically disrupted in 10 volumes of cold PBS, centrifuged at 3,000 g, and the cell supernatants cross-linked with 2% formaldehyde for eight minutes at room temperature. Nuclei were extracted with nuclear extraction buffer and the chromatin was digested with *Hin*dIII on a shaking platform at 37°C overnight. The cross-linked and digested chromatin products were ligated with T4 ligase (100 Weiss units) at 15°C for 12 hours in 7 mL 1 × ligation buffer. Sample quality was measured by semi-qPCR of the *XPB*/*Eccr3* locus, as a control of non-tissue-specific chromatin conformation (see Additional file 6 for primer sequences). Only samples with more than 70% amplification efficiency were used as experimental templates. BAC clones (20 μg) containing *XPB*/*Eccr3* (MRC Geneservice, clone 344-C18), *Pitx2* (CHORI, clone RP24-215O15), *Enpep* (CHORI, clone RP24-172B1) and 3:G3 tested region (CHORI, clone RP23-356C23) were treated in parallel, to generate the control templates.

All primers used (Additional file 6) were designed in an approximately 300 kb region of mouse 3:G3 chromosome spanning the syntenic human 4q25 locus in which the GWAS-identified AF-related variants, *PITX2* and *ENPEP* genes are located. Anchor primers were designed within the *Pitx2ab*, *Pitx2c* and *Enpep* promoter sequences (Figure 3). Three technical replicates of three independent experiments were performed for all sets of test-anchor primers for each tissue. Physical interactions among anchor and test primers, in the experimental and control templates, were measured by qPCR (SYBR® Green) and resulting frequencies were calculated and normalised using the *XPB*/*Eccr3* locus as control [61,62]. Statistical analysis, assuming a normal distribution of data, was performed by one-way analysis of variance (ANOVA) test of Student-Newman-Keuls of the significance of differences among biological samples; the significance threshold was set at *P* <0.05. Error bars represent the SEM for the three biological replicates.

### *In situ* hybridization

*In situ* hybridization was performed on sections of E14.5 embryos essentially as previously described [63]. A pan-*Pitx2* probe was kindly provided by José Luis de la Pompa (CNIC, Madrid, Spain). While this probe recognises all *Pitx2* isoforms, only *Pitx2c* is expressed in the heart [64]. *Hcn4* and *Enpep* dsDNA were amplified by PCR from C57Bl/6 J DNA with primers containing T7 or SP6 RNA polymerase initiation sites (Additional file 6). Sense and anti-sense RNA probes were prepared by PCR using digoxigenin-labelled dsDNA as template Roche (Basel, Switzerland); sense probes were used as negative controls. Embryos used for different probes were processed in parallel in all assays.

## Additional files

Additional file 1:
**Genomic analysis of 4q25.** (A) TAD structure of the 4q25 genomic region (hg19; chr4:110,940,551-113,100,551). Hi-C analysis in three different human cell lines (IMR90, lung fibroblasts; hES, embryonic stem cells; GM12878, lymphoblastoid cells) identifies stable TADs in the gene desert surrounding *PITX2*, one of which includes both the gene promoter and the AF-associated SNPs (highlighted in green). Hi-C data were obtained from http://yuelab.org/hi-c/ [27] (B) Overlapping fragments from AF3. Detailed view of the genomic landscape and evolutionary conservation of the region surrounding fragment AF3 and of the sub-fragments (AF3.1 to AF3.6) used in this study (hg19; chr4:111,706,648-111,715,384). Legend as in Figure 1. Additional file 2:
**Results of transgenic experiments.**
Additional file 3:
**4q25 regulatory elements do not show cell-type specificity.** Activity of 4q25 regulatory elements (AF3, AF3.5 and AF3.6) in mouse HL-1 cardiomyocytes (dark grey), compared to mouse P19 teratocarcinoma (light grey) and human HEK293T embryonic kidney (black) cells. *Nppa* proximal (NppaPE) and *Oct4* distal (Oct4DE) enhancers were used as controls of cell type specificity and the empty pβRFP as a control of basal activity. Data are expressed as mean ± SEM. Statistical significance versus empty pβRFP was calculated with the unpaired Student’s t-test. **P* <0.05, ***P* <0.01 and ****P* <0.001. Additional file 4:
**Assessment of reporter activity in ASE and ASE+AF3.6 transgenic embryos.**
Additional file 5:
**Specificity of 3C interactions with** ***Pitx2*** **and** ***Enpep*** **promoters.** (A) Schematic representation of the interacting regions (2R, 6F and 8F; Figure 3) and of the *Pitx2* and *Enpep* genes showing the location of promoter specific anchor primers (Pitx2ab, Pitx2c, Enpep) and control anchor primers located upstream of *Pitx2ab* (c1), in between *Pitx2ab* and *Pitx2c* (c2), downstream of *Pitx2c* (c3), and upstream (c4) or downstream (c5) of *Enpep*. (B-D) Normalised 3C interactions, expressed as relative crosslinking frequencies (y-axis), between 2R (B), 6F (C) and 8F (D) fragments and controls (c1-c5) and promoters (Pitx2ab, Pitx2c and Enpep), in atria (red) and ventricles (blue). In each graph, the highest crosslinking frequency values were set to 1. Statistical significance was assessed following one-way ANOVA test of Student-Newman-Keuls. ***P* <0.01, ****P* <0.001. Error bars represent ± SEM. Additional file 6:
**Primers used in this study.**

## Abbreviations

3C: chromosome conformation capture
AF: atrial fibrillation
ASE: asymmetric enhancer
bp: base pair
E: embryonic day
GFP: green fluorescent protein
GWAS: genome-wide association studies
HEK: human embryonic kidney
kb: kilobase
LD: linkage disequilibrium
Mb: megabase
mRFP: monomeric red fluorescent protein
PBS: phosphate-buffered saline
SAN: sinoatrial node
SEM: standard error of the mean
SNP: single nucleotide polymorphism
TAD: topologically associated domain
