# Supplementary figures and images for: Long-range regulatory interactions at the 4q25 atrial fibrillation risk locus involve PITX2c and ENPEP
Source: BMC Biol. 2015 Apr 17;13:26. doi: 10.1186/s12915-015-0138-0 (PMC4416339; doi:10.1186/s12915-015-0138-0)

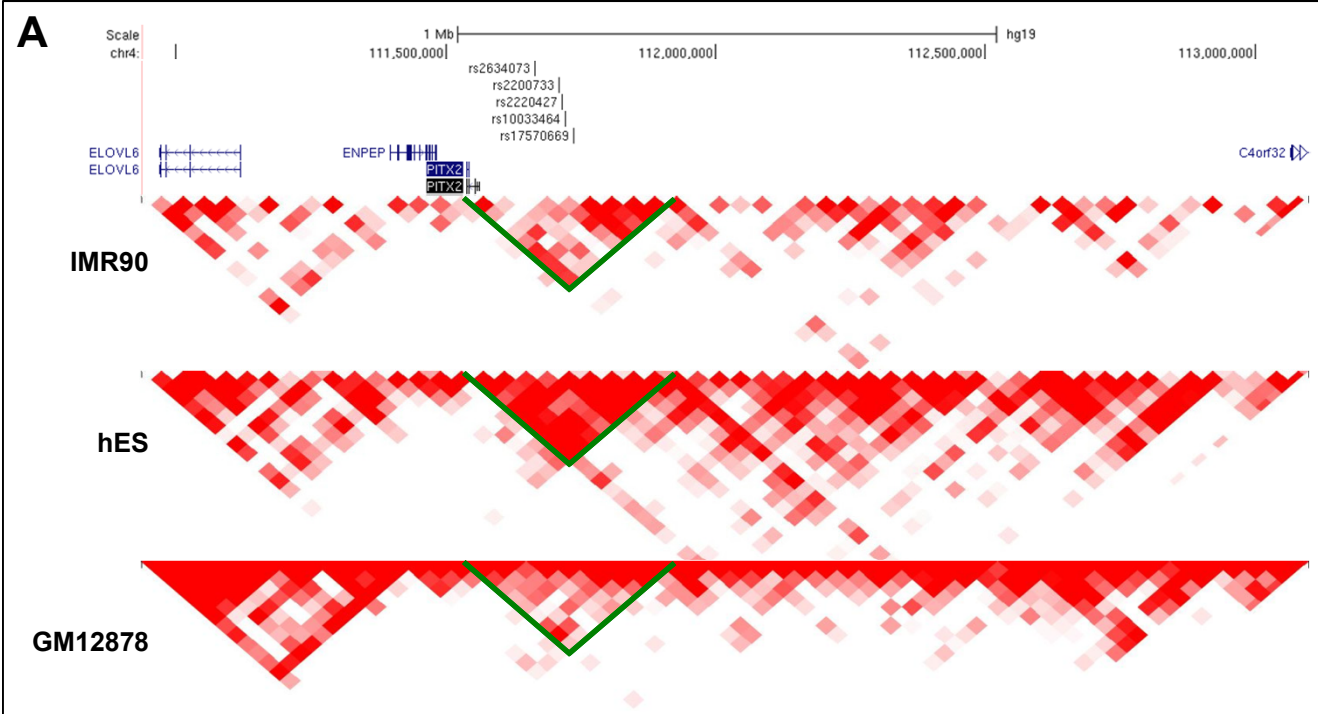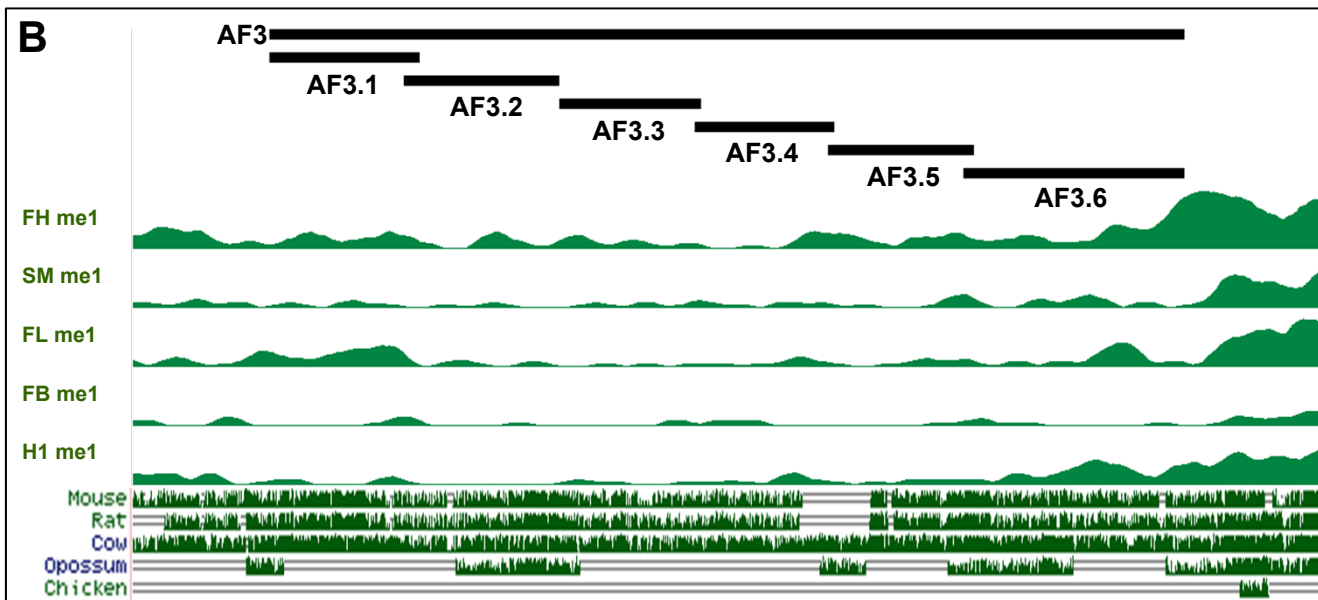

Supplement: Additional file 1: — Genomic analysis of 4q25. (A) TAD structure of the 4q25 genomic region (hg19; chr4:110,940,551-113,100,551). Hi-C analysis in three different human cell lines (IMR90, lung fibroblasts; hES, embryonic stem cells; GM12878, lymphoblastoid cells) identifies stable TADs in the gene desert surrounding PITX2, one of which includes both the gene promoter and the AF-associated SNPs (highlighted in green). Hi-C data were obtained from http://yuelab.org/hi-c/ [27] (B) Overlapping fragments from AF3. Detailed view of the genomic landscape and evolutionary conservation of the region surrounding fragment AF3 and of the sub-fragments (AF3.1 to AF3.6) used in this study (hg19; chr4:111,706,648-111,715,384). Legend as in Figure 1. [file 12915_2015_138_MOESM1_ESM.pdf]

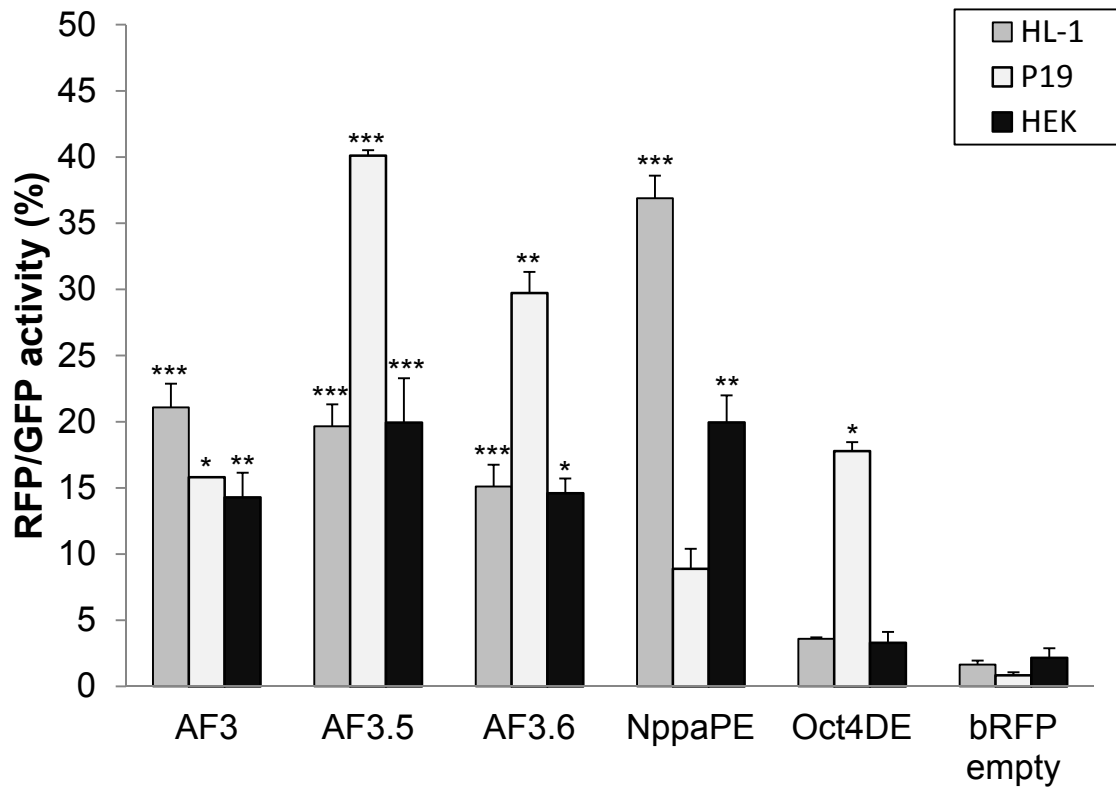

Supplement: Additional file 3: — 4q25 regulatory elements do not show cell-type specificity. Activity of 4q25 regulatory elements (AF3, AF3.5 and AF3.6) in mouse HL-1 cardiomyocytes (dark grey), compared to mouse P19 teratocarcinoma (light grey) and human HEK293T embryonic kidney (black) cells. Nppa proximal (NppaPE) and Oct4 distal (Oct4DE) enhancers were used as controls of cell type specificity and the empty pβRFP as a control of basal activity. Data are expressed as mean ± SEM. Statistical significance versus empty pβRFP was calculated with the unpaired Student’s t-test. *P <0.05, **P <0.01 and ***P <0.001. [file 12915_2015_138_MOESM3_ESM.pdf]

**A**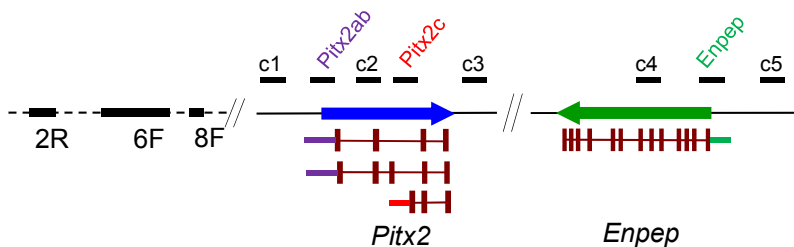**B**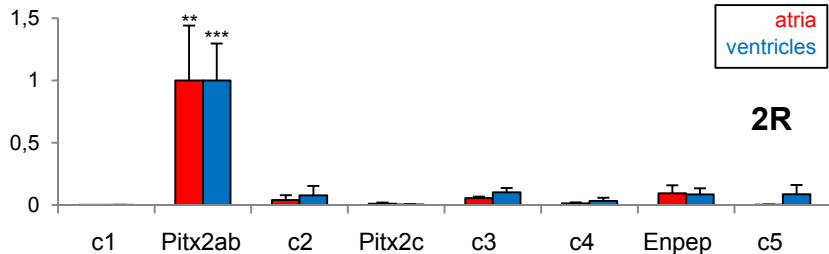**C**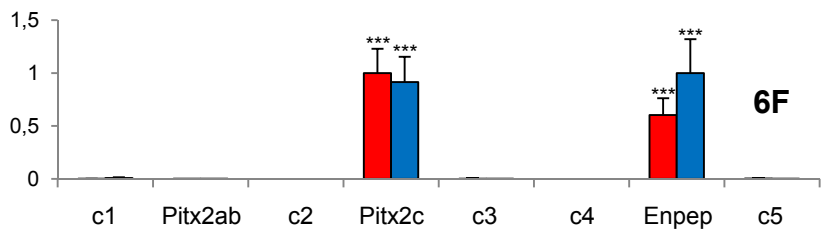**D**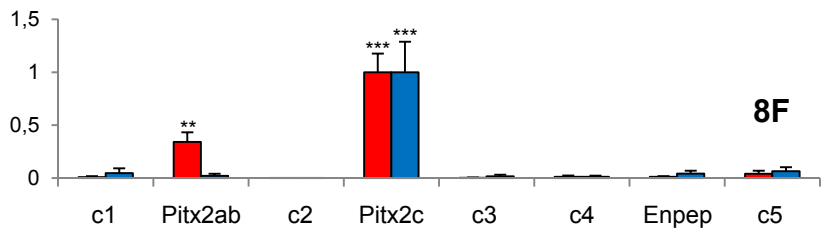

Supplement: Additional file 5: — Specificity of 3C interactions with Pitx2 and Enpep promoters. (A) Schematic representation of the interacting regions (2R, 6F and 8F; Figure 3) and of the Pitx2 and Enpep genes showing the location of promoter specific anchor primers (Pitx2ab, Pitx2c, Enpep) and control anchor primers located upstream of Pitx2ab (c1), in between Pitx2ab and Pitx2c (c2), downstream of Pitx2c (c3), and upstream (c4) or downstream (c5) of Enpep. (B-D) Normalised 3C interactions, expressed as relative crosslinking frequencies (y-axis), between 2R (B), 6F (C) and 8F (D) fragments and controls (c1-c5) and promoters (Pitx2ab, Pitx2c and Enpep), in atria (red) and ventricles (blue). In each graph, the highest crosslinking frequency values were set to 1. Statistical significance was assessed following one-way ANOVA test of Student-Newman-Keuls. **P <0.01, ***P <0.001. Error bars represent ± SEM. [file 12915_2015_138_MOESM5_ESM.pdf]
