# Supplementary material for: Long-range regulatory interactions at the 4q25 atrial fibrillation risk locus involve PITX2c and ENPEP
Source: BMC Biol. 2015 Apr 17;13:26. doi: 10.1186/s12915-015-0138-0 (PMC4416339; doi:10.1186/s12915-015-0138-0)
Supplement: Additional file 2: — Results of transgenic experiments. [file 12915_2015_138_MOESM2_ESM.pdf]

Additional file 2. Results of transgenic experiments.

fragment	# lacZ+	# tg	% lacZ+	# embryos	% tg
AF3	5	11	45	37	30
AF3 (newborn heart)	0	6	0	43	14
AF4	0	9	0	41	22
AF5	0	13	0	45	29
AF7	1	21	5	131	16
AF3.5	2	10	20	110	9
AF3.6	4	17	24	107	16
AF3.5∩3.6	1	17	6	52	33
ASE	5	13	38	46	28
ASE + AF3.6	10	22	45	99	22
