# Supplementary material for: Long-range regulatory interactions at the 4q25 atrial fibrillation risk locus involve PITX2c and ENPEP
Source: BMC Biol. 2015 Apr 17;13:26. doi: 10.1186/s12915-015-0138-0 (PMC4416339; doi:10.1186/s12915-015-0138-0)
Supplement: Additional file 4: — Assessment of reporter activity in ASE and ASE+AF3.6 transgenic embryos. [file 12915_2015_138_MOESM4_ESM.pdf]

Additional File 4. Assessment of reporter activity in ASE and ASE+AF3.6 transgenic embryos.

	embryo	OFT	AVC	LA	RV/IVS
ASE	#1	-	+	-	-
	#2	+	+	+	+
	#3	+	+	-	+
	#4	+	+	+	+
	#5	-	-	-	-
ASE+AF3.6	#6	+	+	+	+
	#7	-	-	-	-
	#8	-	+	+	+
	#9	+	+	+	-
	#10	-	-	-	-
	#11	-	+	+	+
	#12	-	-	-	-
	#13	+	+	+	+
	#14	-	+	+	-
	#15	-	+	+	-

OFT, outflow tract; AVC, atrioventricular canal; LA, left atrium; RV/IVS, right ventricle/interventricular septum.
