# Supplementary material for: Long-range regulatory interactions at the 4q25 atrial fibrillation risk locus involve PITX2c and ENPEP
Source: BMC Biol. 2015 Apr 17;13:26. doi: 10.1186/s12915-015-0138-0 (PMC4416339; doi:10.1186/s12915-015-0138-0)
Supplement: Additional file 6: — Primers used in this study. [file 12915_2015_138_MOESM6_ESM.pdf]

Additional File 6. Primers used in this study.

**4q25 fragments**	
AF1-Forward	GCAATTCTGTGTTAATATAAGAG
AF1-Reverse	GGCCATCAGTAAATCACATTT
AF2-Forward	GCTAAGCTCGCCCCAAGC
AF2-Reverse	GCTAGTATCTTGATGACTTTAT
AF3-Forward	GCAAGAACCAACTAGGGCCA
AF3-Reverse	ATGGAGAATGTAAGTTTGGAGC
AF4-Forward	GCACTGGCTGGATTCATACTTT
AF4-Reverse	ACTATTTGATAGCCTCAGAGC
AF5-Forward	GCAGGGAACATAGGTAAGCTT
AF5-Reverse	ACATATGCAGAGGTAGAATTGC
AF6-Forward	GCCCAAAACACCTCCAAGT
AF6-Reverse	GGGATAACAAATCTGAAAGC
AF7-Forward	GCTGCAAGATAGTATCAATTAAGC
AF7-Reverse	GCTGTTTGCCTCTTCACAATTC
AF3.1-Forward	GCAAGAACCAACTAGGGCCA
AF3.1-Reverse	CCTGTTTACTTGTTAGAGATAACAGG
AF3.2-Forward	CCAACCCAAATTGACAATTCTTGA
AF3.2-Reverse	CATGCAAGACAGAACTCAACAGC
AF3.3-Forward	CGACTCCCTTTCTGACTTTCAT
AF3.3-Reverse	GACAGTGACTCTCAAACTTTAGC
AF3.4-Forward	GCTCTCCAGGAGGTTTTCATG
AF3.4-Reverse	CGGATCACCAAATCCAAAGGC
AF3.5-Forward	CCAGGAGAATAAGAATTGAGAAG
AF3.5-Reverse	CAGATTAAGGCAAGCTCTCACC
AF3.6-Forward	GGTTGTTTCTGGCTTTAAAAT
AF3.6-Reverse	ATGGAGAATGTAAGTTTGGAGC
AF3.5Δ-Forward	CCAGGAGAATAAGAATTGAGAAG
AF3.5Δ-Reverse	ACATGAAATCTGGAAAGAATAA
AF3.6Δ-Forward	ATCTATTCCAGAGCAAAATCTCT
AF3.6Δ-Reverse	ATGGAGAATGTAAGTTTGGAGC
AF3.5∩3.6-Forward	CCATCATCTGCACAATGAGCA
AF3.5∩3.6-Reverse	CAGATTAAGGCAAGCTCTCAC
ASE-SalI-Forward	*GTCGAC*CGCCCGCTATTCCGCAGCTC*
ASE-XhoI-Reverse	***CTCGAG***CCAACACTCTTTCTCGCCTAGC†

*Underlined italicized sequence corresponds to the SalI recognition site tag.

†Bold italicized sequence corresponds to the XhoI recognition site tag.

**Chromosome conformation capture (3C)**	
XPB/Eccr3-Forward	TGACCTCCACACTCCTGAC
XPB/Eccr3-Reverse	ATGCGCAATTAGAAACTGC
Pitx2ab	CAGAAGCACACATTCCACAGAGC
Pitx2c	GTTGGAGGACGCGTACGCCCAC

Enpep	TCTCCTTGTGGGAAGAGATC
c1	ACCCAACATCAACCATTGCT
c2	CTGACCCTGCGTTCCATACT
c3	AGTGCTTTGAATGAGCTTTGG
c4	TGCATTTGCTCAAGGATTTG
c5	GCTGAGAACCCTGAAGTTGC
Hind-1F	TCACCACTGTTCTTAGCTTGAGTGC
Hind-2R	CCATGAGAGCTTCTTGACTGTTTGG
Hind-3F	CCCATGCTTTCTGTCCTAAACC
Hind-4R	CCATGGTAATGCACATGGAAGG
Hind-5R	GGATTCAAACTCAGACCCTGTT
Hind-6F	TGGCTCAGCACTTCACTCATCC
Hind-7F	TCCTCCTAGCTGGCCTATATTTCTG
Hind-8F	GGCCCACAAAGCTGGGTAGC
Hind-9R	TTGGCATTAGTGTGTCCTTACAAAGC
Hind-10F	AGCTTGTAGACACAATCCTTGC

**Transgenic mouse genotyping**	
Myogenin-Forward	CCAAGTTGGTGTCAAAAGCC
Myogenin-Reverse	CTCTCTGCTTTAAGGAGTCAG
LacZ-Forward	GCGACTTCCAGTTCAACATC
LacZ-Reverse	GATGAGTTTGGACAAACCAC

**In situ hibridization (ISH)**	
Enpep-Forward	<u>*ATTTAGGTGACACTATAGAA*</u>ACCGTCATCCGCTACATCTC*
Enpep-Reverse	***GTAATACGACTCACTATAGGG***ATGGCAATACACCCTGCAA†
Hcn4-Forward	<u>*ATTTAGGTGACACTATAGAA*</u>GCATGATGCTTCTGCTGTGT*
Hcn4-Reverse	***GTAATACGACTCACTATAGGG***TTGAAGTTGTCCACGCTCAG†

*Underlined italicized sequences correspond to the SP6 RNA polymerase initiation site tag.

†Bold italicized sequences correspond to the T7 RNA polymerase initiation site tag.
